# On the Motions and Sounds of the Heart

**Published:** 1830-07-01

**Authors:** 


### On the Motions and Sounds of the Heart. By *D. J. Corrigan*, M.D.

We take credit to ourselves for having been the very first who, in this country, drew the attention of the profession to the immortal discovery of Laennec, auscultation. That this should have been persecuted in the onset by the mob, is no matter of astonishment to those who know human nature, and remember that the savans of the day scouted Harvey and his doctrines. Those who would be wits and those who must be fools are always leagued in the holy war of antipathy to improvement, of hatred to innovation. The sneer, and the laugh, and the stubborn back of prejudice are ever turned against him who endeavours with honest enthusiasm to ameliorate the condition of the human race, or to strike a link from the fetters of ignorance. The men who persecuted the starry Galileo and they who jeered at Laennec and the stethoscope, differed only in opportunities, in casual circumstances, and in the fashion of their coats, for their minds were still the same, a leaven of stupid prejudice, ignorance, and enmity. Truth, however, is always an overmatch for bigotry at last, and the force of the truism was never more decisive than in the history of auscultation in this country. Where are the opposers now?

Echo answers, where!

All gone, all fled; or if a relic of the sacred Bœotian troop is to be found, it is skulking in private mansions, or the bye-parts of public institutions, where each, like Cato, gives his little senate laws.

We cannot but rejoice at this result of a contest where one party contended for truth and for science, the other for the prevalence of lazy and confident error. We should be more or less than men if we did not enjoy a triumph for which we have holpen to fight, if we did not exult in some degree when the eagle of victory flapped his wings upon our banners. No, we are delighted at the issue for the sake of the illustrious dead, for the profession, and for humanity. But we cannot avoid perceiving that the good is not unmixed, and that the consequences of the progress of auscultation will not be in the first instance solely beneficial. Pretenders will start up who will prostitute the stethoscope to their own vile ends, or who, knowing nothing of auscultation, will bring it into odium by their quackeries and blunders. That such will be the case we can plainly foresee, nay, we are sorry to confess that it has been so already. We are acquainted with but too many instances of men, whose only pretensions to auscultation were consummate impudence and unmitigated assurance, pronounce with all the gravity in the world on pectoriloquy, ægophony, and so forth, when they knew not these sounds from the noise of a tin kettle or the braying of one of their own fraternity. The fact is that to turn auscultation to any account it is necessary to be well read in the works of Laennec, to be practically conversant with the ordinary symptoms of diseases, to have seen a fair share of actual cases, and to be accustomed to investigations in morbid anatomy. Unless a practitioner be thus prepared, we really would not recommend him to waste his time upon the stethoscope.

Another evil which is likely to attack the infancy of auscultation, is the mania of the present day for rushing into print. If a man applies a stethoscope half-a-dozen times he fancies he has made some marvellous discovery, and hastens, pleno ore, to communicate his mighty secrets to the public through the medium of some of our lively contemporaries. Is there no such thing as modesty in the world? Does a man suppose that with limited observations for a year or two he is called upon to question, or warranted in running a-muck at the opinions of those who have observed with care on an extended scale for a length of time? Really, common sense would seem to say, no! and yet the common practice is for every dabbler in the use of the stethoscope to doubt and dispute, on all occasions, the opinions of one who knew something about it, M. Laennec. God forbid that we should wish to gag the mouths of our brethren, that we should erect the opinions of any man into the authority of revelation, that we should assert M. Laennec to be infallible, or hold up his statements as never to be questioned. Did we broach such ultra-apostolical tenets, we should merit the bitterest sarcasms ever bestowed on the persecutors of Galileo, or the opponents of improvement. Such is not our aim, but we maintain that those who have hitherto had little experience should be slow to call in question the dicta of those who had much, because such a course will ever breed disorder, encourage error rather than elicit truth, puzzle the inquiring, confound the wavering, and be a by-word and a scorn in the mouths of the malignant. None can pay greater respect than we, to the observations of genuine talent and experience, none can entertain a more thorough contempt for the idle niaiseries of the pert jackanapes, or the pompous nothings of the crafty puffee. With these remarks which are absolutely called for at the present moment we proceed to the memoir of Dr. Corrigan. When we see occasion we shall criticize freely, for the worse the sore the more bold should be the application of the caustic or the bistoury. The author commences with some very just remarks on the great improvement that has taken place of late years in the diagnosis of diseases of the chest.

"Our knowledge of the diseases of the heart, and lungs, has advanced within these late years, with a rapidity unparalleled in the history of medicine. For this we are indebted principally to the labours of Corvisart and Laennec. The light thrown by these writers on diseases of the heart and lungs, and the development by them of symptoms of whose existence there was not, before their time even a suspicion, have given to the physiology of these organs an increased interest; insomuch that inquiries, which, previously might have been attended with little practical advantage, have now become absolutely necessary, and their results of the utmost consequence in enabling us to form correct diagnoses. In the physiology of these organs, that of the heart stands foremost, its motions and sounds presenting perhaps the most remarkable phenomenon in the living body." 152.

It seems that Dr. Corrigan was hardy enough to publish a memoir on the bruit de soufflet in the Lancet, and, what is yet more worthy of admiration, he is sufficiently simple or confident to refer to it. We know not what Dr. Corrigan may think, nor do we particularly care, but this we will say, that such a mode of coming before the public is not calculated to win the ears of the respectable part of the profession, in England. The acquaintance of the costermongers of St. Giles's is not a good passport to society in St. James's, and so such writers as Dr. Corrigan will discover. We have expressed our opinions on this subject with freedom, because we should consider the total suppression of our decided disapprobation, not to use a more harsh expression, as little less than reprehensible hypocrisy. But to business.

All men, be they physiologists or not, are aware that the heart, to use the phrase of our immortal bard, "knocks at the ribs," and most persons believe that the beat of the heart is synchronous with the pulse in the artery at the wrist. The explanation of this impulse of the heart against the side has always been a matter of some difficulty, but anatomists have universally agreed that it takes place during the contraction or systole of the ventricle. The generally received opinion is that which has been thought to emanate from Hunter, and is as follows:—

"'The systole and diastole of the heart simply could not produce such an effect, nor could it have been produced, if it had thrown the blood into a straight tube in the direction of the axis of the left ventricle, as is the case with the ventricles of fish, and some other classes of animals, but by its throwing the blood into a curved tube, viz. the aorta, that artery at its curve endeavours to throw itself into a straight line to increase its capacity, but the aorta being the fixed point against the back, and the heart in some degree loose or pendulous, the influence of its own action is thrown upon itself, and it is tilted forward against the inside of the chest.'" 153.

We have always considered this explanation far-fetched, but our limits will not allow us to enter into physiological discussions at present, and we pass to the more practical points which are involved in the question. Dr. Corrigan objects to Hunter's explanation, and criticises also the opinions of Dr. Bostock* and Mr. Alderson "of Cambridge College,"† but without going into the grounds of his demurral, it is sufficient to say that they are partly satisfactory and partly not. But whilst we participated in the Doctor's repugnance to admit the accuracy of the foregoing explanations of a fact, we were somewhat startled at his positive denial of the fact itself, and his bold declaration, that the impulse and the ventricular contraction are not synchronous at all.

"All physiologists assert, that it is during the contraction of the ventricles, the heart strikes the side. The insufficiency of all explanations, and the errors of most, led me to think that there was probably some mistake in the premises on which all have argued. The first step therefore was, to ascertain whether the position universally assumed, was correct, namely, that it is during the systole of the ventricles the heart strikes the side.

---

* Bostock's Physiology, Vol. 3, p. 398.
† Quarterly Journal of Science, &c. Vol. 18, p. 223.

"The arteries being always full, and fluids being nearly incompressible, it follows that an impulse from the ventricle must be felt in the arterial branches, at the very instant of time of the contraction of the ventricle; that therefore, the pulse indicates precisely the moment of that contraction. The arterial pulse then being exactly synchronous with the contraction of the ventricle, and the striking of the heart against the side being, according to all physiologists, a consequence of that contraction, it follows that the arterial pulse should be felt a moment before the heart strikes the side, or that at the farthest, the impulse against the side, and the pulse, should be synchronous. Is it so? Or are they even synchronous? They are not. I know that my readers will be startled by this assertion, for all physiologists assert that they are; but let the reader before he discredits my assertion that they are *not*, but that the impulse of the heart against the side is anterior to the arterial pulse, place the index finger of his right hand on the point where his own heart beats most strongly, at the same time keeping the thumb or forefinger of the left upon the radial artery of his right hand. When his heart is beating slowly and forcibly, he will perceive distinctly that the first tap is against the ribs, the second from the pulse.

"The *second* tap indicates the precise moment of the contraction of the ventricle; the *first*, the heart's impulse against the side: the contraction of the ventricle is consequently posterior to the impulse of the heart. An effect cannot precede its cause; therefore, the contraction of the ventricle which follows, can not produce the heart's impulse, which has gone before. This experiment on the side and wrist was conclusive in my mind, that the contraction of the ventricles could not be the cause of the heart's impulse, and convinced me that the statements given by all physiologists of the heart's movements were erroneous, and that the position assumed by all, namely, that the heart strikes the side when the ventricles are contracting, was false. Some other cause of the heart's impulse was therefore to be sought." 167.

We like to give authors fair play, and therefore we will lay the whole of Dr. Corrigan's arguments before our readers, before we attempt urging any remarks of our own. The impulsion then and the dull sound attending it, are attributed by our author to the contraction of the auricles, and not to that of the ventricles, as all physiologists and Laennec have concluded. As confirmatory of this view Dr. Corrigan mentions a case in which the "bruit de soufflet" was distinctly anterior to the pulse. He had no doubt that this sound was produced by the rush of blood through a narrow opening into a wider cavity. As it was anterior to the pulse, and synchronous with the impulse, he concluded that the latter was owing to the contraction of the auricle, and that the bruit depended on narrowing of the auriculo-ventricular opening. On dissection it proved to be so. This is the case which is designed to immortalize the Doctor, and to which he refers with that feeling of proud satisfaction, that something which the world can neither give nor take away, experienced by heroes or martyrs on atchieving a great or a glorious deed. However, our author fearing lest "he might have been too much carried away by his opinions," perhaps elated and dizzy at seeing himself blush in Lancet type in company with the X Y Z's, and other ornaments of science that adorn that respectable magazine, determined, like Trajan, to associate others in the empire, who might ease his cares and share his glory. Doctor John C. Ferguson and Doctor Hunt, were the fortunate men selected on the occasion, and with our author constituted the triumvirs, chosen by kind Fate to decide the momentous question in abeyance. Their observations, like themselves, are of a triple nature, and consist, "for the sake of clearness," of first, the experiments made—secondly, the facts observed and conclusions arrived at —and thirdly, a testing of the new views, by the litmus paper of pathology.

" Our first object was to examine the heart in the living animal. A rabbit was selected, and before proceeding with the experiment, the stethoscope was applied. Both sounds of the heart could be distinctly heard. The right side of the chest was opened, the mediastinum being left uninjured. Respiration went freely on, little blood was lost; and we obtained a view of the heart in action, far superior to what we could have anticipated. The animal lived in this state for about twenty minutes, sufficient time for accurate examination. Immediately after the operation, and when the heart was first brought into view, its motions were very rapid and tumultuous, but in a few moments they became less frequent, and more regular, and the movements of the different parts plainly distinguishable: first, the contraction of the auricles—second, the contraction of the ventricles—then, the pause. At each contraction of the auricles the heart came forward, the ventricles being dilated in every direction, and driven downwards and forwards; at each contraction of the ventricles the heart retired into the chest. The contractions of auricles and ventricles were quick; but it is impossible to describe the rapidity with which the contraction of the ventricle followed that of the auricle. When the heart was beating violently, their succession was so rapid, that the eye could scarcely distinguish between the two contractions." 169.

The experimenters could observe no such motion as the heart being tilted up against the ribs; but it first applied itself to the parietes of the chest by a small surface almost midway between the base and the apex which quickly increased in extent.

" The stethoscope was now placed on the left side of the sternum, and while one of us listened to the sounds of the heart's action, and tapped with his finger at each impulse and dull sound, the others marked by the sight the contractions of the heart. The tap indicating the impulse and dull sound, came after each pause, and synchronous with the contractions of the auricles, the ventricles being at the same instant dilated and propelled forward, the appendices of the auricles at the same time retiring.—Another now took the stethoscope; the experiment was conducted in the same way, and with a similar result. These experiments were repeated six times on rabbits, and again, on a larger animal, and invariably with the same results." 170.

The motions of the heart in warm-blooded animals under pain are so rapid as to occasion a difficulty in their analysis by the naked eye. The cold-blooded reptile, a frog for instance, is free from this inconvenience. The following criticisms on Dr. Bostock's assertions respecting the heart's action in this animal do not seem to us to be strictly just, but we give them as they are, with the observations grafted on them.

" Doctor Bostock, describing the motion of the heart in a cold blooded animal, uses the following words.—' For a short space of time the heart lies at rest, and suffers itself to be distended with blood, then it is suddenly seen to rise up on its basis, to shorten its fibres, and to expel its contents.'* We may observe first, that there is in this description, a contradiction both to his own assertions in another part of his work, and a refutation of his theory of the heart's impulse. Doctor Bostock makes in this description, the rising up of the heart anterior to the contraction of the ventricles. He says in another place, ' I may without impropriety assert, that the beating is felt *not* at the instant

* Bostock's Physiology, vol. i. p. 346.

when the ventricle *begins to contract*, but when the contraction has produced its effect in filling the arch of the aorta.' In the first quotation the heart, according to Doctor Bostock, *first* rises up, that is, beats, *then* expels its contents; in the second passage the heart *first* fills the arch of the aorta, that is, expels its contents, and then beats. He asserts with Senac and Hunter, that the filling of the arch of the aorta by the contraction of the ventricle, is the cause of the heart's rising and giving an impulse; yet in the description just quoted, he makes the rising of the heart anterior to the filling of the arch. Doctor Bostock's description is, however, not only at variance with his own assertions and theory, but it is even quite erroneous in point of fact. This, the inspection of the heart in the living frog clearly showed. Having removed the inferior portion of the sternum, and thus brought the heart clearly into view, the following phenomena were observed. The heart did not suffer itself to be distended with blood, as Doctor Bostock states. The blood was thrown into it by the auricle contracting with great energy. It did not rise up on its basis; but was dilated and driven downwards and forwards by the blood expelled from the auricle: and finally, as the ventricle contracted, the heart retired from the surface, being deepest in the chest, at the moment when the contraction was as its utmost.

"The heart of a frog is very large, compared with the size of the animal; its movements are very slow, and its parietes although strong, are almost transparent. These circumstances, but above all, the transparency of the walls of the organ give the greatest certainty to observations on the actions of the heart in this animal; the presence or absence of blood in the ventricle being marked, not alone by the increase or decrease of size, but also in the most beautiful manner by the change of colour. The heart is quite pale when the ventricle is contracted or empty, a deep rich purple, when it is dilated or full of blood. This change of colour was an additional test of the accuracy of our observations, which we did not possess in warm blooded animals. When the auricle was distended it came fully into view; when it contracted, it did so with great energy, retiring quickly from our sight. At the same instant the ventricle swelling, being distended with blood (as shown by its sudden change from extreme paleness to a rich purple colour,) was impelled with some force against the finger. The contraction of the ventricle followed quickly upon its dilatation. It diminished itself in every direction, bringing its sides together, and its apex towards the base, and as it contracted, retired until its perfect paleness proved, that it had expelled all its blood; the heart at the moment, when the contraction was at its height, being deepest in the chest. Repetitions of this experiment confirmed our observations in every particular." 173.

Having thus concluded the detail of the experiments, we are presented with the facts ascertained, and they are these.

"1mo. The auricles contract first.

"2ndo. The ventricles, second.

"3tio. Then the pause or state of rest.

"4to. The contraction of the ventricles is rapid, and follows quick as can be conceived after that of the auricles.

"5to. The contraction of the auricles is comparatively slow.

"6to. The heart strikes the side, or, gives its impulse, when the auricles contract.

"7mo. The heart retires from the side when the ventricles contract.

"8vo. The beat of the heart is produced, not by a tilting up of the point of the organ as hitherto described, but by its swelling and coming against the ribs, in consequence of the impulse given by the rush of blood from the auricle." 173.

We now proceed to the stethoscopic portion of the investigation, the sounds produced by the heart's action. Laennec has announced that the first sound heard on applying the ear, which is synchronous with the impulse against the side is that of the ventricle, the sound immediately succeeding the former, than which it is louder and clearer, is that of the auricle. Such are the statements of Laennec, but if Dr. Corrigan be right in denying that the stroke against the chest is produced by the ventricle, Laennec's explanation of the nature of the sounds must fall to the ground of course.

"To ascertain the relation which the sound bears to the pulse, we selected from very extensive dispensary practice, those patients, who happened to have remarkably slow pulses. We also examined repeatedly and carefully the heart's action in the horse; which animal offered peculiar facilities for our purpose. The heart, when in health, beats only forty in the minute, and the sounds are distinct. Though perfectly satisfied in our own minds, with the result of these observations, still we looked for further evidence. Each of us selected, from among his friends not in the profession, a person on whose delicacy of ear and accuracy of observation he could rely. Of these, one was a gentleman who has been blind for some years, a man, however, of the highest mental powers. It is needless to remark, that, from this peculiar circumstance, loss of sight, his other senses have become extremely acute. The persons thus selected were carefully kept in ignorance of either our own views, or those entertained by others. After they had to their satisfaction distinguished the two sounds and the impulse, through the stethoscope, they were instructed to lay a hand on the pulse, and note the order in which each phenomenon seemed to them to occur.

"The opinions expressed by these persons were as follow.—'The impulse and dull sound came before the pulse. The dull sound had terminated when the pulse struck the finger. The short sound came exceedingly quick *after* the pulse. The first sound was long; the second short, not half the length of the first; and there was a short interval between the two sounds.' These observations, made by our non-medical friends, we hold to be peculiarly valuable; they are those of persons unprejudiced, made without a knowledge of any theory: they are the records of facts, the persons who recounted them not knowing for what purpose they were to be used. On the dull sound and impulse preceding the striking of the pulse, there was not amongst them even a hesitation; all declared at once that the impulse and dull sound preceded the pulse, and by a well marked interval. On the termination of the dull sound there was a slight difference, one asserting it to have ended when the pulse struck the finger; *another supposing it and the pulse to finish together*. When the heart beat quickly, the dull sound and pulse seemed to terminate together; but in the horse, and in slowly beating hearts, the pulse terminated the long sound, as if with a blow, or, as the tap of a finger terminates the vibrations of a glass." 177.

Consequently Dr. Corrigan maintains that the first long sound is that of the contraction of the auricle and filling of the ventricle, which exactly reverses Laennec's statement and the commonly received opinion. In illustration of his opinions our author refers to the particulars of a case of contraction of the left auriculo-ventricular opening, but we think it affords him but sorry support, and by-and-bye we may advert to it again.

Again with respect to the second sound; Dr. Corrigan contents himself with saying that it cannot depend upon the auricle, for that contracts first. From an experiment which we have not room to detail, but which really appears to us inconclusive and objectionable in every respect, Dr. Corrigan was led to conclude, that it was produced by the contraction of the left ventricle and the impulsion and collision of the two opposite sides against each other. To be sure the best physiologists believe that the ventricle does not entirely empty itself, and that no such collision takes place, but Dr. Corrigan answers this "assumption" by saying that "it is totally unsupported by proof, contrary to reason, and directly contradicted by experiment."

Our author next applies the new lights to the éclaircissement of the diagnostic sounds produced by alterations of the heart. Laennec has laid down as a general law, that extent and loudness of sound without impulsion, are the signs of passive dilatation; impulse with little or no sound, those of simple hypertrophy of the ventricle; and a strong impulse and loud sound, the marks of hypertrophy and dilatation. Of the general accuracy of these signs no stethoscopical observer of disease of the heart can entertain a doubt, and accordingly Dr. Corrigan, feeling that to dispute them would be dangerous, endeavours to explain them on his own views. We must say that the attempt is but a scurvy one. There is one item which, if true, would upset the Doctor's auricular views, the co-existence of much impulsion with hypertrophy of the ventricle; for if the auricle produced the impulse, why should it be greater when the ventricle became hypertrophic? Dr. C. perceiving that disentanglement would be difficult, boldly cuts the knot by asserting that increase of impulse is not a *constant* attendant on hypertrophic ventricles, and by insinuating that it is not a common one. In order to give the coup de grace to the overthrow of the unfortunate ventriculists, he cites the following case from Bertin.

"We shall give," writes the Doctor, "a case from Bertin, so satisfactory, as to convince the most sceptical, giving those parts of it which bear upon our subject, in his own words. The case is headed, '*Hypertrophy of the left auricle*, of the interventricular septum, and of the columnæ carneæ of the right ventricle with dilatation of the left cavities, *narrowing, or rather, disappearance of the cavity of the right ventricle*, and *ramolissement of the walls of the left*, a little thickened towards the point.'* After having given a lengthened detail of the usual symptoms of heart disease, with which it is unnecessary to trouble the reader, he adds, 'his pulse was full, free, and *vibrating*, (vibrant.) *The beatings of the heart were felt with violence over almost the whole chest.*'—'Autopsy; the pericardium was very adherent to the heart, particularly on its anterior surface. The heart was very large, and rounded, presenting no appearance of its apex. *The left auricle was enormously dilated, and its walls had acquired a thickness of three lines.* The left ventricle had acquired a similar dilatation, and its cavity was so much increased, that it contained eight ounces of fluid; but *its walls were not thickened, unless at the inferior part and point. They were soft and easily torn:* the interventricular septum was more than an inch thick through its whole extent. Its tissue possessed no more consistence than the other parts of the walls. The right auricle was natural, but the right ventricle was *wasted*, and possessed, at most, not more than one-fifth of the volume of the left. The columnæ carneæ equalled the size of a writing pen, and were adherent to one another, occupying the cavity of the ventricle, so that during life, the blood could only filter through their meshes. The mitral valve was cartilaginous on its edges; the aortic valves were similarly affected in their middle." 196.

To this case Dr. Corrigan attaches much value, and so do we, but for a precisely opposite reason. The interventricular septum was an inch in thickness, the left ventricle was greatly dilated and hypertrophied at the inferior part and point, and the pericardium was adherent. If these pathological conditions were not sufficient to cause "the beatings of the heart to be felt with violence over almost the whole chest," even without the hypertrophy and dilatation of the left auricle, but more certainly with it, then will we renounce auscultation, forget our practical experience, and whistle our case-book down the stream for ever and for aye. Is not Dr. Corrigan

* Traité des maladies du cœur, &c. p. 334.

aware that adhesions of the pericardium will produce a most extensive and violent action of the heart, with little or no hypertrophy and dilatation of auricle or ventricle? We have witnessed many such cases, and so have all men of experience not warped and twisted by theory. So completely is this the fact, that physicians of eminence, unacquainted with auscultation, have looked on an extensive convulsion of the chest, with violent action of the heart, as the characteristic sign of adhesions of the pericardium; and if there have been a rheumatic history, they are right in nine cases out of ten. Unfortunately this is not the only instance in the present memoir, of Dr. Corrigan's displaying a large stock of theory and credulity, or both, and a small one of practical information.

"It may be said, are there not many cases related of hypertrophied and dilated ventricles with increased impulse, and without hypertrophied auricles? We are well aware of this, but we must take leave to doubt the accuracy of these reports. Nor is this to be wondered at, when we remember the material errors already pointed out in so accurate an observer as Laennec; when we reflect that the important part, which the auricles play in the actions of the heart, has never been pointed out until the present view—when we find that the auricles have engaged so little attention that Bertin and Laennec have not said one word by which to guide observers to judge of their hypertrophy, while they have been very minute on the relative proportions of the parietes of the ventricles. These writers have laid it down as an established fact, that increased impulse is invariably caused by hypertrophy of the ventricles. Their statement has been taken as true, and hence it has naturally arisen, that while attention was eagerly turned to the state of the ventricles, that of the auricles has been passed over as of little consequence." 199.

We agree with Dr. Corrigan, that sufficient attention has not been paid to the auricles, nay, we will go farther than he, and assert that the great mass of practitioners would not know a hypertrophied auricle if they saw it. There is a lamentable want of precise information on the subject of diseases of the heart, for we have frequently seen physicians, and of some repute, pronounce that a heart was "a little dilated," when the left-ventricle was hypertrophied to double its volume. Many men, indeed, can never perceive disease in the heart, unless it be actually converted into bone or positively as large as a bullock's. Show them a heart thick or thin, hypertrophied or flabby, and if the fact be not too palpable and gross to allow the bye-standers to keep their countenances on the occasion, these gentlemen will say with much sang-froid, "Oh, it is healthy enough, it is a well-proportioned heart, for the size of the patient." We shall conclude by giving our author's own summary of his opinions.

"Of the Motions of the Heart.

1mo. The contraction of the auricles (comparatively slow,) takes place first.

2ndo. The contraction of the ventricles extremely rapid, follows quick as thought upon that of the auricles.

3tio. The pause.

4to. The impulse of the heart against the side does not take place during the contraction of the ventricles, but in their dilatations.

5to. The impulse against the side is caused, not by the contraction of the ventricles, but by the contraction of the auricles, being dependant on the force with which the auricles send their blood into the ventricles.

6to. When the auricles contract, the ventricles are dilated, and the heart comes forward.

7mo. When the ventricles contract, the heart retires.

Of the Sounds.

1mo. The first sound is caused by the rush of blood from the auricles into the dilating ventricles; not by the contraction of the ventricles as hitherto taught.

2ndo. The second sound is caused, not by the contraction of the auricles, the falling back of the heart, or the action of the valves, but by the striking together of the internal surfaces of the ventricle.

Of the Rhythm.

1mo. The impulse and long sound come first, and are synchronous.

2ndo. The pulse.

3tio. The second or short sound." 201.

Having now given as full an account of Dr. Corrigan's paper as could be done without actually transferring it servilely from his page to our's, it only remains for us to offer a few remarks upon the doctrines it contains. Our readers, we are sure, will acquit us of prostrating our intellects before the dogmas of authority, or belonging to the ranks of those who worship only what Bentham has quaintly denominated "the wisdom of our ancestors." If we disagree then with Dr. Corrigan it is not merely because what he promulgates is new, but because we conscientiously believe that it is not true. The pivot on which all his doctrines turn is the question of the impulse of the heart against the side being consentaneous with the pulse or not. The profession have always believed that it is, Dr. Corrigan maintains that it is not, and one would think that on this point any one might satisfy himself. Since commencing the present review we have examined a considerable number of patients in a large hospital, and the result of our observations is in decided opposition to that of Dr. Corrigan's;—in fact, in the majority of cases the impulse was so synchronous with the pulse that neither we nor a demonstrator of anatomy who accompanied us could detect any want of accordance. In some few instances indeed the impulse and first sound were slightly anterior to the pulse at the wrist, but even here the interval was extremely minute and the pulse still seemed to be more in accordance with the first than the second sound. We have frequently remarked, and no doubt others have made the observation, that the pulse at the wrist has something approaching to a double beat, which confers upon it a vibratory character. What this may be owing to we do not know, but it is always when the first cardiac sound is long and the impulse more or less considerable. In this kind of pulse the second slight beat may indeed be thought to correspond with the second sound of the heart, and in point of time does so, but we question whether it be essentially connected with it. So much for the correspondence between the impulse and first sound with the pulse at the wrist, a correspondence which our senses appear to confirm.

Allowing, however, that the stroke against the side were a little anterior to the pulse, we do not conceive that the non-dependence of the one upon the other must necessarily follow. If the heart of a dog, yet palpitating, be placed upon a table, the apex is observed to be lifted up at each contraction of the empty ventricles.* Surely this action must be in some degree anterior to the complete or nearly complete expulsion of the contents of the

* Mayo's Physiology, 1st edit. p. 68.

ventricle, and surely also the pulse must feel the effect of the last drop of fluid expelled from the heart. Dr. Corrigan maintains, to suit his theory, that the ventricle empties itself completely, and he maintains with singular inconsistency, to suit his theory also, that the arterial pulse should even be felt a moment *before* the heart strikes the side; as if forsooth it were merely the first impulse communicated to the blood that is felt at the extremity of the system, whilst the ventricle goes on contracting to the uttermost without producing any further effect upon the pulse! In the observations of Dr. Corrigan's blind friend it is remarked "that the short (second) sound came exceedingly quick *after* the pulse, and his other acquaintance "supposed the *first sound and the pulse to terminate together!* Was there ever any thing so unfortunate for the Doctor! His very pupils trained with stethoscope and touch for the occasion, arise and prophesy against him!

With regard to the experiment on the rabbit we can only say in the newspaper phraseology, it would be important if true. We do not mean to breathe a whisper against Dr. Corrigan's veracity, God forbid! But we know the fallacies of experiments, the slips of the human mind, and we likewise know that theory, like Love, is blind.

The last point requiring attention is the evidence afforded by pathology. Dr. Corrigan asserts that impulsion is not the guage of hypertrophy of the ventricle; we assert as a general rule, that it is, and that it no more indicates hypertrophy of the auricle than it helps to foretel an eclipse. We assert, and from experience, that the diagnosis of hypertrophy of the ventricle is generally very simple indeed, because it is almost invariably accompanied with a dull first sound, approaching even to the bruit de soufflet and increased impulsion. Who does not know a *cardiac pulse*, and who does not know that it is accompanied with the morbid impulsion and alteration of the first sound? Have men found hypertrophic auricles on these occasions? Alas, Dr. Corrigan, they have not. In our remarks we speak from what we have seen and from many cases, not from what we think, and from a few.

Dr. Corrigan refers to two cases of contraction of the left auriculo-ventricular opening attended with the bruit de soufflet as confirmatory of his views. He asks how could the first sound be attended with the *bruit* under such circumstances, if the ventricle produced it? Dr. Corrigan must surely be aware that there is such a thing as regurgitation of the blood, that all physiologists allow the ventricles to drive some blood back into the auricles in a state of health, and that in cases of rigid contraction of the auriculo-ventricular opening, where the valves can no longer close it effectually, such regurgitation must be still more likely to take place. If Dr. Corrigan is aware of these circumstances he will not press his question very closely, and if he was not we trust he will feel obliged to us for mentioning them. But farther than this, we assert that contraction of the left auriculo-ventricular opening is not necessarily attended with any bruit de soufflet whatever, nay, more, that in the majority of cases which we have witnessed it has been absent. For proof of this we refer to the last Number of this Journal in which we recorded five or six cases of this disease.

Our narrow limits compel us to bring these criticisms to a close, and we can only say that we have done more justice to our author than ourselves in the space we have allotted to the expression of his and our sentiments.

We have only to state in conclusion, that though quite accessible to conviction, it will require much more than Dr. Corrigan has yet adduced to shake our belief in our present opinions or diminish our scepticism respecting his.

